# Identification of a PadR-type regulator essential for intracellular pathogenesis of *Burkholderia pseudomallei*

**DOI:** 10.1038/s41598-021-89852-7

**Published:** 2021-05-17

**Authors:** Ian A. McMillan, Michael H. Norris, Jan Zarzycki-Siek, Yun Heacock-Kang, Zhenxin Sun, Bradley R. Borlee, Tung T. Hoang

**Affiliations:** 1 School of Life Sciences, University of Hawaiʻi at Mānoa, Honolulu, Hawaiʻi USA; 2 Present Address: Department of Geography and Emerging Pathogens Institute, University of Florida, Gainesville, FL USA; 3 Department of Microbiology, Immunology, and Pathology, Colorado State University, Fort Collins, CO USA

**Keywords:** Infection, Bacteriology, Transcriptional regulatory elements

## Abstract

*Burkholderia pseudomallei* (*Bp*) is the causative agent of melioidosis, a disease endemic to the tropics. Melioidosis manifests in various ways ranging from acute skin lesions to pneumonia and, in rare cases, infection of the central nervous system. *Bp* is a facultative intracellular pathogen and it can infect various cell types. The *Bp* intracellular lifecycle has been partially elucidated and is highly complex. Herein, we have identified a transcriptional regulator, BP1026B_II1198, that is differentially expressed as *Bp* transits through host cells. A deletion mutant of BP1026B_II1198 was attenuated in RAW264.7 cell and BALB/c mouse infection. To further characterize the function of this transcriptional regulator, we endeavored to determine the regulon of BP1026B_II1198. RNA-seq analysis showed the global picture of genes regulated while ChIP-seq analysis identified two specific BP1026B_II1198 binding regions on chromosome II. We investigated the transposon mutants of these genes controlled by BP1026B_II1198 and confirmed that these genes contribute to pathogenesis in RAW264.7 murine macrophage cells. Taken together, the data presented here shed light on the regulon of BP1026B_II1198 and its role during intracellular infection and highlights an integral portion of the highly complex regulation network of *Bp* during host infection.

## Introduction

Bacteria move through various niches responding and reacting to each specific environment they encounter. For this to occur, bacteria must transduce environmental cues through complex signaling pathways that lead to transcription of specific factors required for survival in each situation. These networks are highly complex and require the coordination of many components. Coordinated response to environmental signals often require transcription factors that can modulate RNA polymerase binding to a promoter region, leading to the activation or repression of an operon or gene^1^. Binding of transcription factors to specific DNA sequences is often controlled through interactions with small ligands, other proteins, and/or by covalent modification^1–3^. Control of transcription factors can be mediated through several mechanisms including turnover, sequestration, and synthesis that can also be coordinated by other transcription factors^1^. Additionally, a transcription factor can bind to several promoter regions, which in turn can be controlled by several transcription factors on a local and/or global scale^1^. Taken together, this highlights the highly complex system that bacteria use to coordinate a transcriptional response to each environment they confront. Pathogens, in particular, encounter many environments during the course of an infection, requiring them to have a well-coordinated regulation system in place.

*Burkholderia pseudomallei* (*Bp*) is a facultative intracellular pathogen that causes the disease melioidosis. *Bp* is endemic in tropical regions with increasing prevalence due to better diagnostics and rising awareness, having a predicted global mortality rate of 54 percent^4^. *Bp* encounters many different environments as it infects most tissues of the human body, leading to the formation of localized abscesses, bacteremia, septic shock, and sometimes death^5,6^. While infecting a cell, *Bp* has a dynamic lifecycle that can be broken down into multiple stages: attachment to the host cell, host cell entry, vacuole escape, cytoplasmic replication, and protrusion towards neighboring cells culminating with the spread of infection^7^. In the vacuole, the *Burkholderia* secretion apparatus (T3SS_Bsa_), a type III secretion system, must be expressed to allow entry into the host cell cytoplasm^8^. Within the cytoplasm, *Bp* can use its secondary flagella or polymerize host cell actin using bacterial expressed BimA to move freely^8,9^. Eventually, *Bp* protrudes towards neighboring cells and uses a virulence-associated type VI secretion system (T6SS-5) to fuse host cell membranes, forming multinucleated giant cells (MNGCs)^10–13^. Although we understand the general flow of the *Bp* intracellular lifecycle, there is much more we do not know about how this pathogen transits through a host and causes the many forms of the disease we cumulatively call melioidosis.

To cause such a wide array of clinical manifestations and be able to transit through the intracellular environment with such precision, *Bp* requires many virulence factors, some of which are described above. As recently demonstrated, *Bp* goes through drastic transcriptional changes, with 1,953 genes showing unique patterns of expression during the intracellular infection process^7^. This finding suggests that *Bp* has a highly complex regulatory network that is required to coordinate the differential expression of these genes during the progression of infection, from the initial stage of attachment to eventual protrusion towards neighboring cells. The regulation of *Bp* virulence mechanisms during intracellular infection has been studied to a small extent and mainly focuses on the control of T3SS_Bsa,_ T6SS-5, and BimA. The transcriptional regulator BprP regulates expression of the structural components of the T3SS_Bsa_ and another transcriptional regulator BsaN^14,15^. BsaN with its chaperone, BicA, regulate expression of type III effectors and accessory proteins, the two-component sensor-regulator VirAG that is responsible for downstream expression of T6SS-5, BimA, and BprC, a transcriptional regulator essential for T6SS-5 expression^14–16^. Beyond the specific control of characterized virulence pathways, a LysR-type transcriptional regulator GvmR (globally acting virulence and metabolism regulator) was identified to control a broad network of genes involved in virulence and metabolism, highlighting the tie between expression of virulence factors and nutrient limitation within the host^17^. This study advances the understanding of *Bp* virulence regulation by describing a new transcriptional regulator that is differentially expressed throughout the intracellular lifecycle and required for complete pathogenesis.

## Results

### BP1026B_II1198 is differentially regulated as *Bp* transits through the host cell

**Figure 1:**
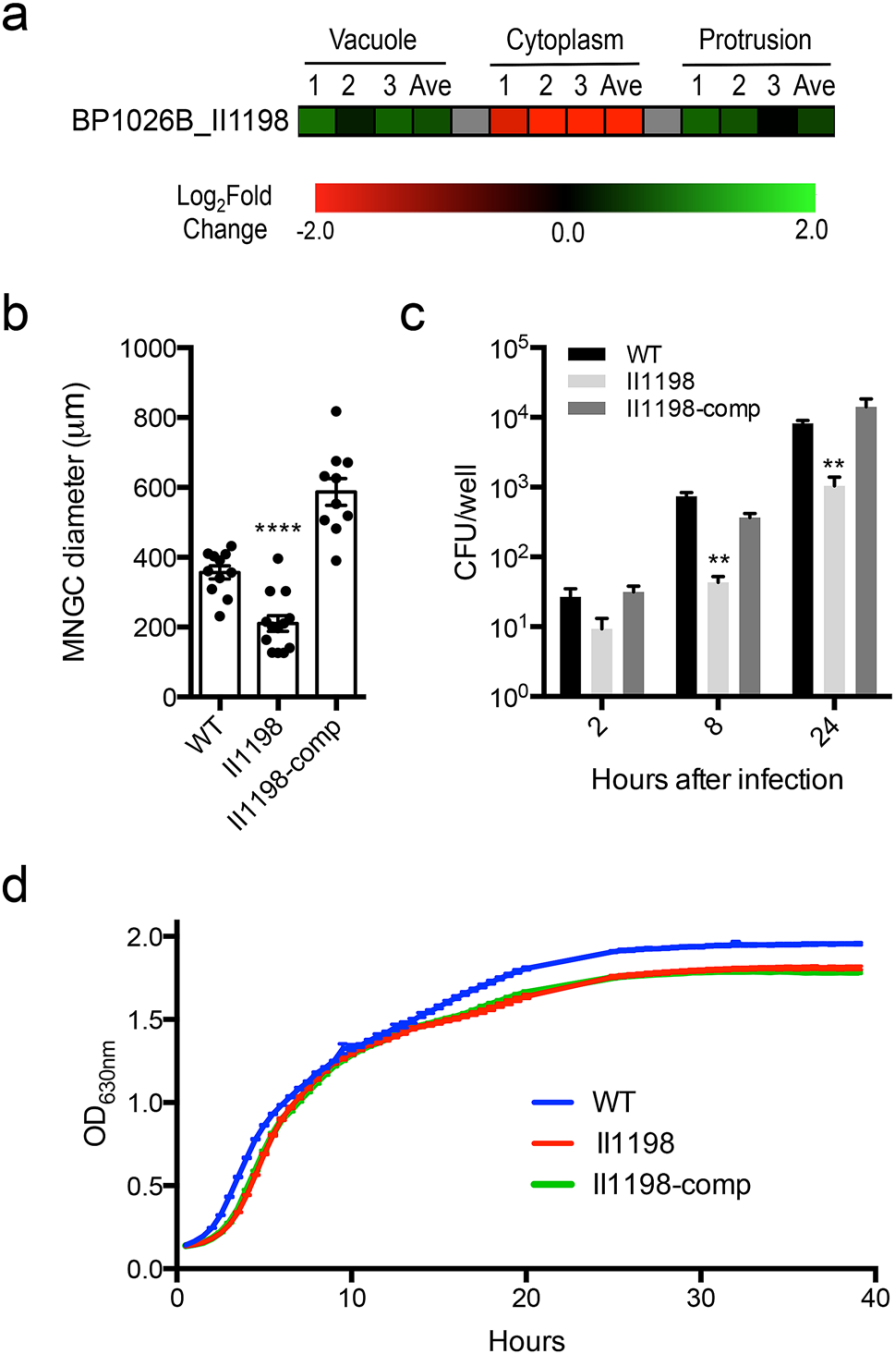
BP1026B_II1198 is differentially regulated during cell infection and critical for intracellular survival. (**a)** Single cell ‘transitome’ analysis^7^ shows that BP1026B_II1198 is up-regulated in the vacuole and protrusion while down-regulated in the cytoplasm during infection of RAW264.7 cells. Boxes represent three biological replicates and an average (Ave)^7^. (**b**) A mutant in BP1026B_II1198 shows a reduced ability to form wildtype level MNGCs. When the mutant is complemented it recovers beyond wildtype level of MNGC size. Individual MNGC diameters are shown as dots. (**c**) The BP1026B_II1198 mutant shows a significant reduction in intracellular replication at 8 and 24 h after infection compared to wildtype *Bp* 1026b. Complementation of the BP1026B_II1198 mutant recovers the intracellular replication to wildtype levels. (**d**) The BP1026B_II1198 mutant and complement grow to similar levels of wildtype *Bp* 1026b in vitro. Data in bar graphs and growth curve represent means ± s.e.m and analyzed via unpaired t-test. P values presented are as follows: ** p < 0.01, ****p < 0.0001.

To understand how intracellular pathogenesis of *Bp* is controlled, we first have to identify transcriptional regulators that control virulence mechanisms during intracellular infection. We identified BP1026B_II1198, a PadR-like transcriptional regulator that is differentially regulated during the *Bp* intracellular ‘transitome’^7^. BP1026B_II1198 shows similarities to PadR transcriptional regulators with an N-terminal winged helix-like DNA binding domain (Pfam: PF03551) and a C-terminal virulence activator alpha domain (Pfam: PF10400)^18,19^. BP1026B_II1198 is expressed while *Bp* is initially internalized within endocytic vesicles and later during protrusion as *Bp* spreads toward neighboring cells (Fig. 1a). In contrast, BP1026B_II1198 is significantly down-regulated while *Bp* replicates within the cytoplasm (Fig. 1a). This suggested that BP1026B_II1198 could play a regulatory role during the *Bp* intracellular lifecycle.

### A BP1026B_II1198 mutant is defective in MNGC formation and intracellular replication

To further investigate the role of BP1026B_II1198, a deletion mutant was created using modified knockout-recombineering^20^. This mutant was used to infect RAW264.7 cells to monitor MNGC formation and intracellular replication. The BP1026B_II1198 mutant strain showed a significant decrease in its ability to form MNGCs, marked by a 41% decrease in average MNGC diameter at 24 h post infection (hpi) (Fig. 1b). Complementation of the BP1026B_II1198 mutant restored this mutation beyond wildtype levels (Fig. 1b). RAW264.7 cells infected with the BP1026B_II1198 mutant also showed a significant decrease in intracellular replication at 8 and 24 hpi further suggesting that this transcriptional regulator is important for intracellular pathogenesis (Fig. 1c). Complementation of the BP1026B_II1198 mutant nullified this defect allowing wildtype levels of intracellular replication (Fig. 1c). The BP1026B_II1198 mutant showed a seven percent reduction in growth compared to wildtype *Bp *in vitro (Fig. 1d). This slight decrease in growth indicates that the reductions in MNGC formation and intracellular replication are likely due to defects in pathogenesis rather than in vitro fitness.

### BP1026B_II1198 is required for complete pathogenesis in vivo

**Figure 2:**
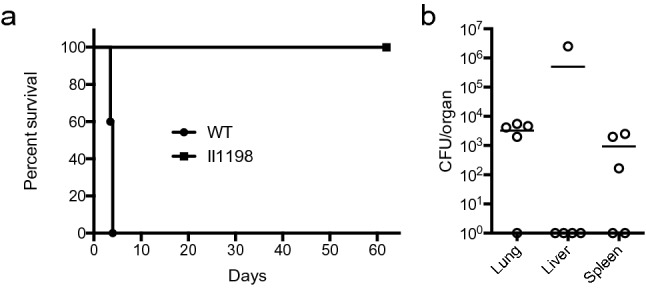
BP1026B_II1198 is attenuated in the BALB/c mouse model. (**a**) Mice infected with wildtype *Bp* 1026b (n = 5) did not survive beyond day four post challenge while mice infected with the BP1026B_II1198 mutant (n = 5) were able to survive for the duration of the study (62 days). Comparison of both experimental groups by Log-rank (Mantel-Cox) test shows a significant difference (P = 0.0031) in survival. Mice were challenged with a lethal dose (4500 CFU/mouse) via the intranasal route. (**b**) Lungs, livers, and spleens of the surviving mice were harvested, homogenized, and plated revealing persistent infection within the lungs of four mice, liver of one mouse, and spleen of three mice. No detectable bacteria were found in the lungs of one mouse, livers of four mice, and spleens of two mice (circles along x-axis).

Beyond cell culture infection, we wanted to assess the importance of BP1026B_II1198 during an in vivo infection. BALB/c mice were infected via the intranasal route with a lethal dose (4,500 CFU/mouse) of wildtype *Bp* 1026b and an equivalent dose of the BP1026B_II1198 mutant strain. Within four days of the initiation of infection, all mice infected with wildtype *Bp* 1026b showed significant signs of acute murine melioidosis resulting in 100% lethality (Fig. 2a). In contrast, mice infected with the BP1026B_II1198 mutant survived for the duration of the study (62 days) indicating that this gene is important for in vivo pathogenesis of *Bp* (Fig. 2a). Even though mice challenged with the BP1026B_II1198 mutant showed no clinical symptoms at day 62 post infection, there was persistent infection in the lungs and spleens of the majority of the mice and one mouse showing high levels of infection within the liver (Fig. 2b). This level of persistence demonstrates a shift in the behavior of *Bp* in the absence of the BP1026B_II1198 gene. This result indicates that BP1026B_II1198 is important for in vivo* Bp* pathogenesis.

### BP1026B_II1198 regulates hypothetical proteins, secondary metabolite biosynthesis, extracellular matrix, efflux, and type VI secretion

**Figure 3:**
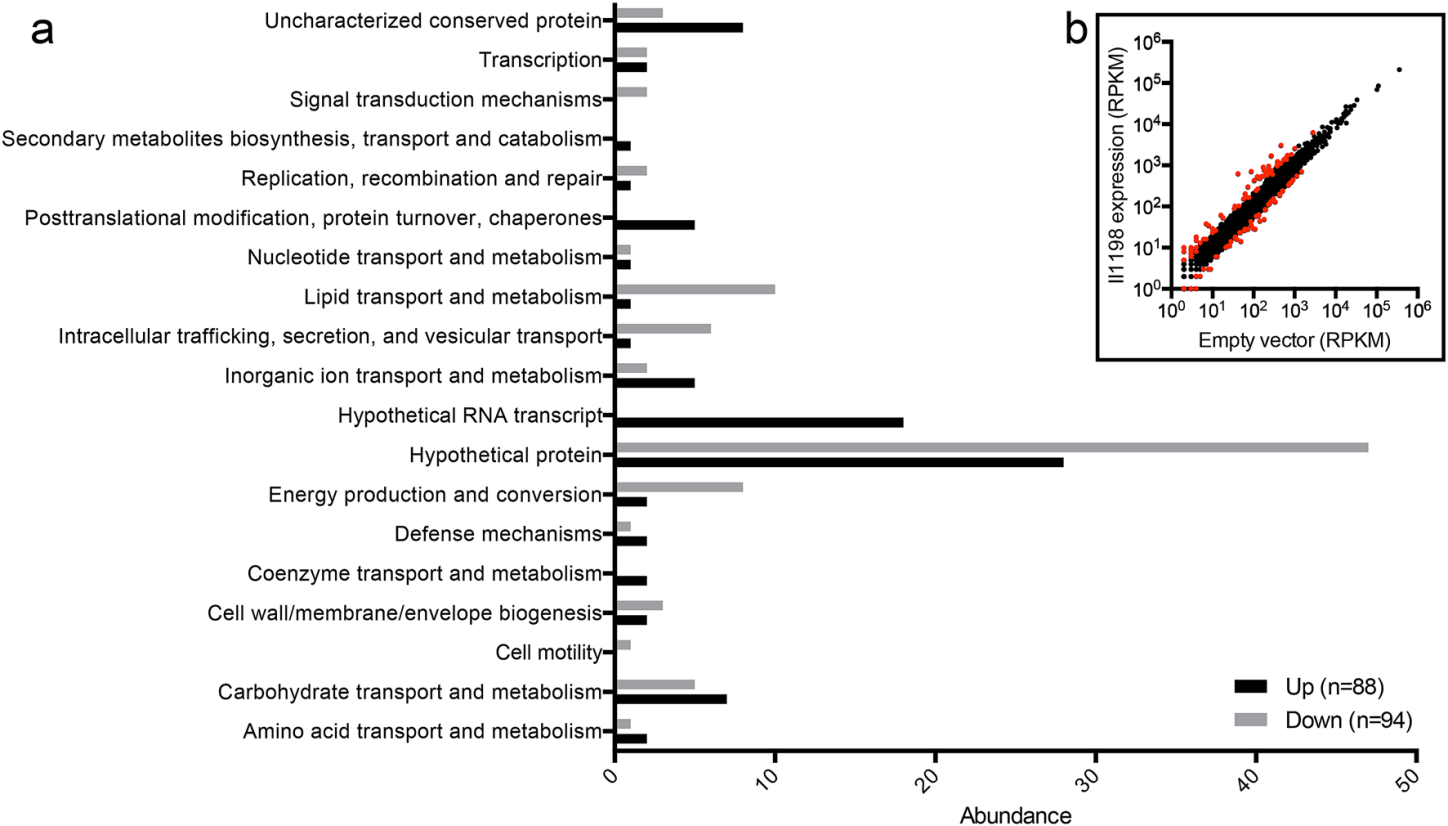
Hypothetical proteins are disproportionately controlled by BP1026B_II1198. (**a**) Genes with a q-value < 0.01 and a log_2_FC ≤ -1 or ≥ 1 were analyzed by COG functional predictions. There are 88 genes that are up-regulated and 94 genes that are down-regulated by BP1026B_II1198. Hypothetical proteins, hypothetical RNA transcripts and uncharacterized proteins represent 79.8% of the genes controlled by BP1026B_II1198. Of these unknown proteins 68% are up-regulated and 32% are down-regulated. (**b**) Scatter plot showing distribution of all genes present in RNA-seq analysis. Genes with a q-value < 0.01 and a log_2_FC ≤ -1 or ≥ 1 are highlighted in red.

To better understand how BP1026B_II1198 controls *Bp* pathogenesis, we expressed BP1026B_II1198 *in trans* and compared the mRNA profile to a control strain with an empty vector control via RNA-seq analysis. Samples were analyzed using Rockhopper^21^ identifying differential regulation of a subset of *Bp* genes confirming that BP1026B_II1198 is a transcriptional regulator (Fig. 3b). Genes with a q-value < 0.01 and a log_2_ fold-change (FC) ≥ 1 or ≤ -1 were selected for further analysis (Fig. 3b, Table S1). There were 88 genes that were up-regulated (log_2_FC ≥ 1) in the presence of BP1026B_II1198 while 94 genes were down-regulated (log_2_FC ≤ -1) (Fig. 3). Genes with a log_2_FC ≥ 1 or ≤ -1 were analyzed by COG functional predictions revealing an overabundance (57.1%) of genes annotated as encoding hypothetical proteins, hypothetical RNA transcripts, or uncharacterized proteins that are controlled by BP1026B_II1198 (Fig. 3a). Of these proteins of unknown function, 54 genes are up-regulated and 50 genes down-regulated by BP1026B_II1198 (Fig. 3a). While this analysis shows the individual genes that are controlled, it is valuable to identify larger regions of the genome that are controlled by BP1026B_II1198 to gain another perspective on its transcriptional landscape. When we analyze the entire RNA-seq data set (Fig. 3b) using WoPPER^22^, some additional pathways that are responsible for secondary metabolite biosynthesis, components of the extracellular polymeric matrix, and multidrug efflux appear to be controlled by BP1026B_II1198 (Figs. S1 and S2).

Two nonribosomal peptide synthetase regions that produce the siderophores malleobactin^23^ and pyochelin^24^ are up-regulated by BP1026B_II1198 in cluster 12 (Fig. S1) and cluster 7 (Fig. S2), respectively, suggesting the involvement of this regulator in iron acquisition during infection. BP1026B_II1198 also up-regulates the polyketide synthase cluster producing another siderophore, malleilactone (Cluster 2, Fig. S2). Malleilactone is involved in the pathogenesis of *Caenorhabditis elegans* and *Dictyostelium discoideum*^25^. Beyond controlling secondary metabolite pathways, BP1026B_II1198 also down-regulates components of the extracellular polymeric matrix tied to contact dependent growth inhibition, BcpA (Cluster 18, Fig. S2)^26–28^. The genes encoding the RND efflux pump, BpeEF-OprC, responsible for multidrug efflux and clinical resistance to antimicrobials^29,30^, are up-regulated in the presence of BP1026B_II1198 (Cluster 3, Fig. S2). Mutation of the *bpeEF-oprC* efflux pump in clinical and environmental isolates of *Bp* showed increased susceptibility to trimethoprim, a component of trimethoprim-sulfamethoxazole that is used in oral eradication phase antimicrobial therapy of melioidosis^30^*.* Although we see low levels of up-regulation of *bpeEF-oprC*, we suspect that the up-regulation is indirect and could involve other regulatory factors. BP1026B_II1198 also up-regulates a region of the genome that includes the type VI secretion system (T6SS-5) that is required for complete pathogenesis and allows intercellular spread and MNGC formation (Cluster 15, Fig. S2)^8,11,31^. This supports the observation that the BP1026B_II1198 mutant showed reduced MNGC formation in RAW264.7 cells (Fig. 1b). The regulation of known pathways and the numerous hypothetical proteins indicates that the BP1026B_II1198 regulation network is tied to predicted and confirmed pathogenic processes, emphasizing the role of BP1026B_II1198 during pathogenesis.

### Specific genes controlled by BP1026B_II1198 have potential role during pathogenesis

**Figure 4:**
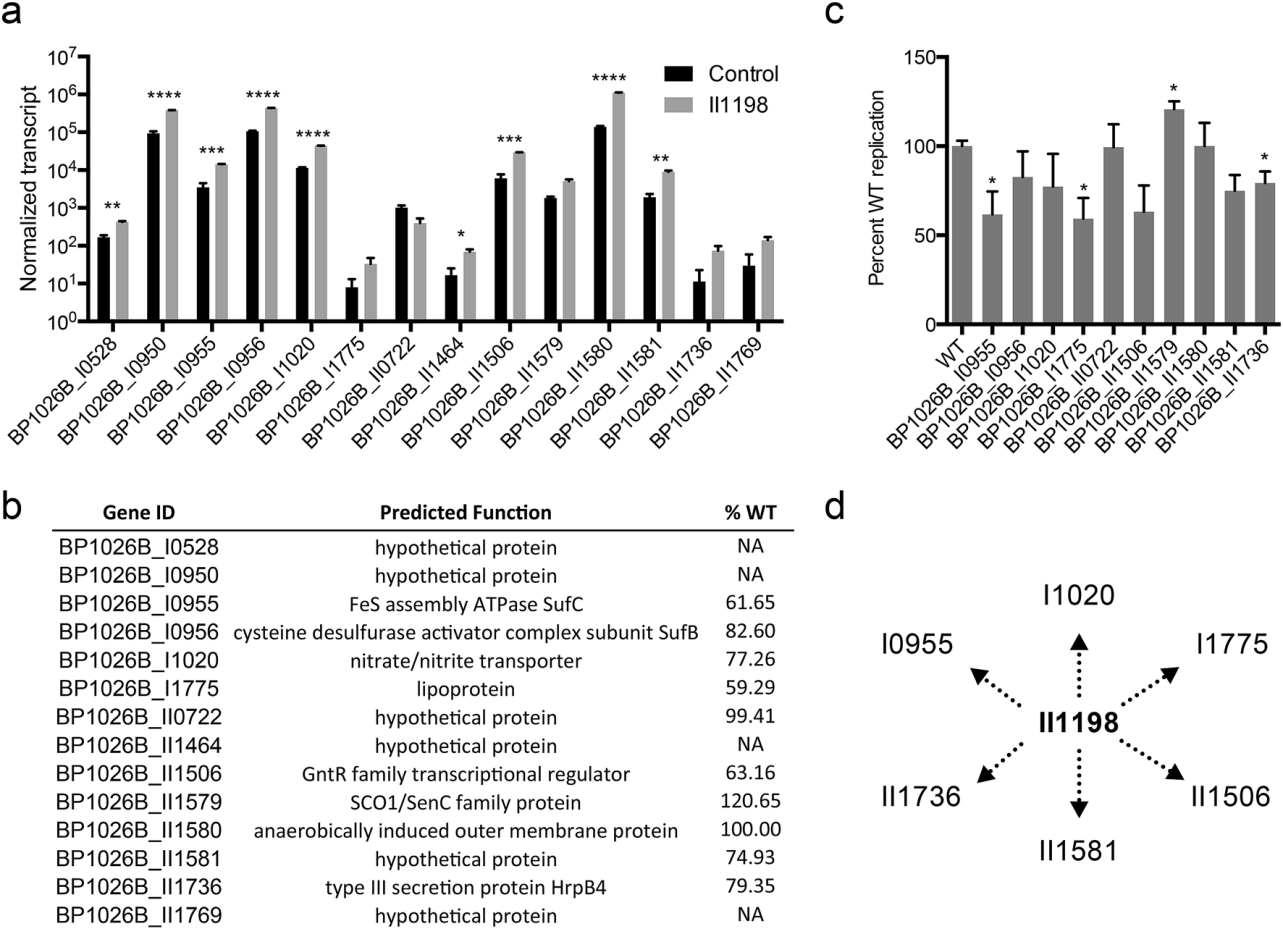
Genes highly regulated by BP1026B_II1198 show involvement during *Bp* intracellular survival. (**a**) Normalized transcripts of genes showing a log_2_ FC ≤ -2 or ≥ 2 in the presence of BP1026B_II1198 compared to an empty vector control. (**b**) Predicted functions of genes controlled by BP1026B_II1198. Percent of wildtype replication (%WT) in RAW264.7 cells is shown 24 hpi. Several transposon mutants were not available in the sequence-defined transposon mutant library of *Bp* 1026b (NA). (**c**) Transposon mutants present in the sequence defined transposon mutant library of *Bp* 1026b were analyzed for the ability to replicate in RAW264.7 murine macrophages for 24 h. Intracellular replication represented as a percent of wildtype 24 hpi and the average of triplicates represented in (**b**). (**d**) Schematic diagram showing genes involved in *Bp* intracellular survival (replicating < 80% wildtype) by indirect control of BP1026B_II1198. Data in bar graphs represent means ± s.e.m and analyzed via unpaired t-test. P values presented are as follows: *p < 0.05, **p < 0.01, ***p < 0.001, ****p < 0.0001.

To further our understanding of this regulation network, we investigated individual genes that are highly regulated by BP1026B_II1198 with a log_2_FC ≥ 2 or ≤ -2 (Fig. 4a). BP1026B_I0955 and BP1026B_I0956 are significantly up-regulated by BP1026B_II1198 with log_2_FCs of 2.16 and 2.03, respectively (Fig. 4a). These genes encode putative SufC and SufB subunits of the SufBCD complex responsible for the assembly of iron-sulfur (Fe-S) clusters in proteins^32^. Genes encoding NifU and SufD are additional components of this Fe-S cluster assembly pathway that are also up-regulated to log_2_FCs of 1.06 and 1.58 (Table S1). Another redox related protein, BP1026B_II1579, is up-regulated to a log_2_FC of 2 and encodes a SCO1/SenC family protein predicted to be involved in the assembly of cytochrome c oxidase (Fig. 4a). Interestingly, BP1026B_II1198 also up-regulates a putative nitrate transporter, BP1026B_I1020 (*narK-1*), with a log_2_FC of 2.09, and a potential *Burkholderia* specific nitric oxide forming nitrite reductase, BP1026B_II1580 (*nirK*), with a log_2_FC of 2.99 (Fig. 4a)^33^. Other components involved in nitrogen metabolism, including *narG* and *narK-2,* are also up-regulated (log_2_FC 1.93 and 1.42) by BP1026B_II1198 (Table S1). The regulation of these pathways by BP1026B_II1198 suggests that they play a role in *Bp* intracellular survival and virulence similar to how nitrate transport/reduction and nitrite reduction have been tied to virulence in *Pseudomonas aeruginosa*^34,35^. In addition to the control of metabolic pathways, BP1026B_II1198 also up-regulates BP1026B_II1736, a type III secretion protein HrpB4 (Fig. 4a).

Many genes that showed large alterations in expression by BP1026B_II1198 are annotated as hypothetical proteins or have no known function (Fig. 3a). BP1026B_I1775 is predicted to be a lipoprotein that shows high amino acid identity to CpaD, a putative pilus assembly protein, in environmental bacteria and is up-regulated by BP1026B_II1198 (Fig. 4a). A GntR family transcriptional regulator, BP1026B_II1506 is also up-regulated and could cause downstream changes in gene expression leading to variations in pathogenesis and/or intracellular survival (Fig. 4a). BP1026B_I0528, BP1026B_I0950, BP1026B_II1464, BP1026B_II1581, and BP1026B_II1769 are hypothetical proteins that are up-regulated by BP1026B_II1198 while BP1026B_II0722 is a hypothetical protein that is down-regulated (Fig. 4a).

### Mutant analysis identifies potential virulence-associated genes controlled by BP1026B_II1198

To test the role these genes play in pathogenesis, we used mutants present in the sequence-defined transposon mutant library of *Bp* 1026b for intracellular replication analysis in RAW264.7 cells^36^. Genes with normalized transcripts showing a log_2_ FC ≤ -2 or ≥ 2 in the presence of BP1026B_II1198 compared to the empty vector control were selected for analysis (Fig. 4a). Four of these genes, BP1026B_I0528, BP1026B_I0950, BP1026B_II1464, and BP1026B_II1769, had no mutants in the library and were not analyzed. Monolayers of RAW264.7 cells were infected with each transposon mutant present in the library and the level of intracellular replication determined at 24 h post infection (hpi). Transposon mutants of BP1026B_II0722 and BP1026B_II1580 replicate to wildtype levels (99–100%) after 24 h of infection indicating that these genes have no singular pathogenic function (Fig. 4b-c). In contrast, transposon mutants in BP1026B_II0956, BP1026B_I1020, BP1026B_II1581, and BP1026B_II1736 replicate between 74.9% and 82.6% of wildtype implying these genes have a small role in pathogenesis (Fig. 4b,c). BP1026B_II1736 is part of T3SS-2 that has been previously tied to virulence in *S. lycopersicum* but the T3SS-2 mutant showed wildtype levels of cytotoxicity in THP-1 cells^37^. This indicates that BP1026B_II1736 and the T3SS-2 cluster could be involved in intracellular survival but does not contribute to cytotoxicity. Mutants of BP1026B_I0956 and BP1026B_I1020, components of the SUF pathway and nitrate transport/reduction, exhibited decreased pathogenesis indicating that these genes and pathways are important for *Bp* intracellular survival (Fig. 4b,c). Transposon mutants of BP1026B_I0955, BP1026B_I1775, and BP1026B_II1506 showed the greatest decrease in intracellular survival, replicating at 61.7%, 59.3%, and 63.2% of wildtype, respectively (Fig. 4b,c). Finally, the SCO1/SenC family protein, BP1026B_II1579, replicates at 120.65% of wildtype indicating that protein is involved in reducing the intracellular survival of wildtype *Bp* within host cells (Fig. 4b,c). Transposon mutants showing a major decrease in intracellular replication were able to replicate at wildtype levels when grown in vitro indicating that the defects identified during RAW264.7 cell infection are not due to decreased fitness (Fig. S3a). This data suggests that BP1026B_II1198 controls several factors that contribute to intracellular pathogenesis to varying degrees (Fig. 4d).

### BP1026B_II1198 directly binds to two intergenic regions on chromosome II

**Figure 5:**
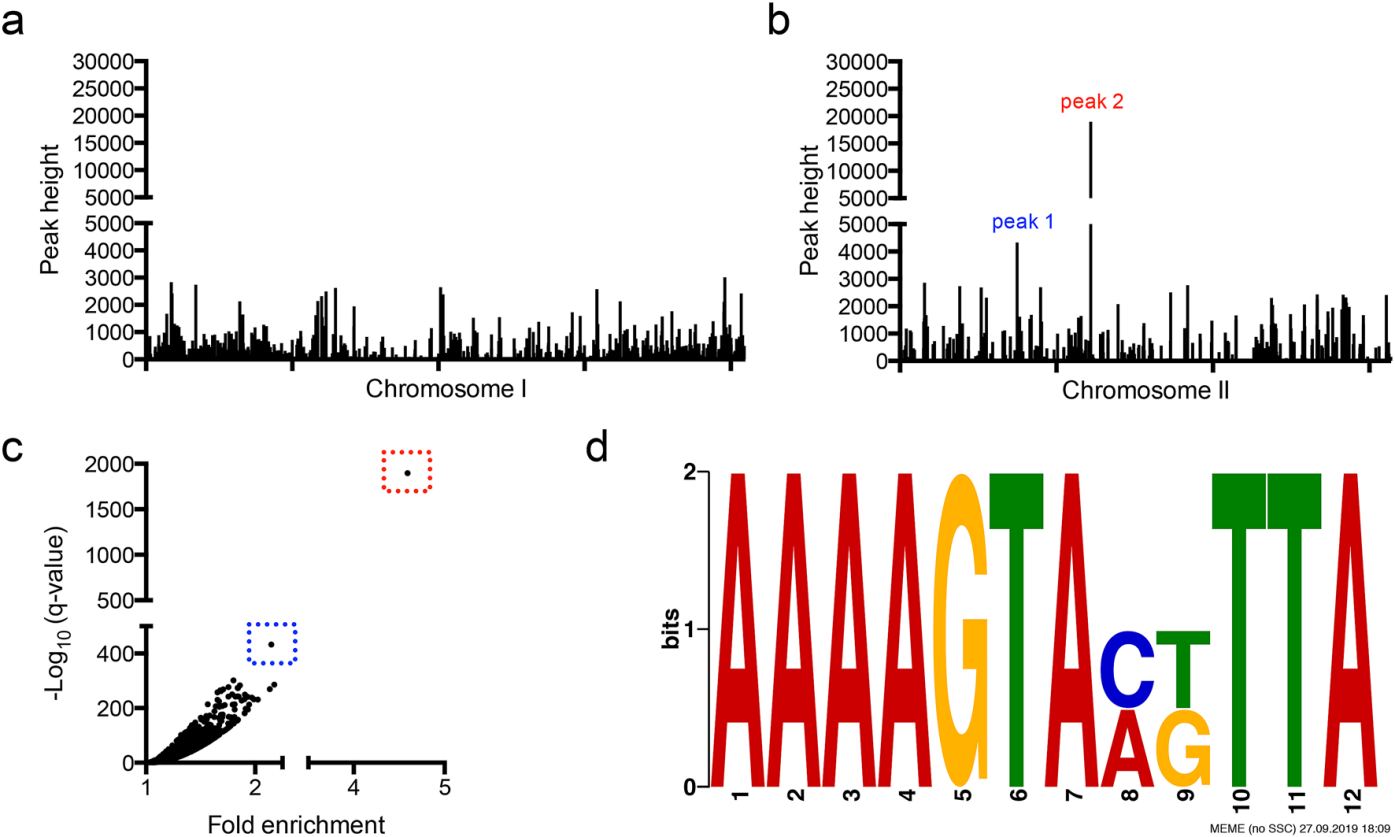
ChIP-seq identifies two regions of BP1026B_II1198 binding on chromosome II. Peaks identified using MACS2^76^ by comparison to an empty vector negative control are plotted by peak height and genome position on chromosome I (a) and chromosome II (**b**). (**b**) Two peaks were identified on chromosome II and are labeled as peak 1 in blue and peak 2 in red. (**c**) The two identified peaks are further isolated from background when compared by statistical (-log_10_ q-value) and fold enrichment. Blue box shows peak 1 and red box shows peak 2. (**d**) Potential binding motif identified by analysis of peak 1 and peak 2 nucleotide sequences by MEME^38^. Each peak contains the same orientation of the potential binding motif.

**Figure 6:**
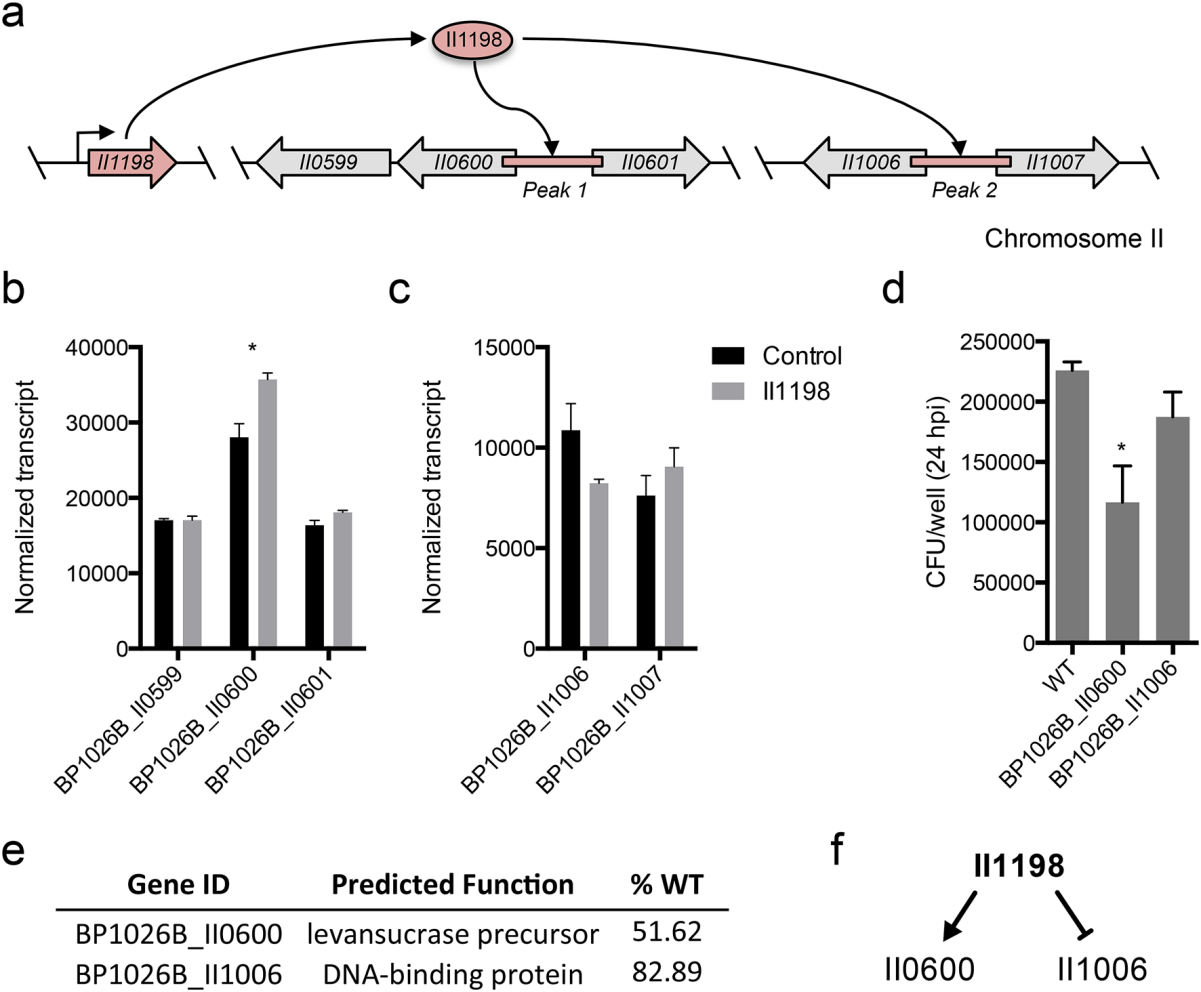
BP1026B_II1198 has direct transcriptional control of genes involved in pathogenesis of RAW264.7 murine macrophages. (**a**) Schematic representation of the direct regulation network of BP1026B_II1198. BP1026B_II1198 binds to an intergenic region (peak 1) between BP1026B_II0600 and BP1026B_II0601 and an intergenic region (peak 2) between BP1026B_II1006 and BP1026B_II1007. (**b**) Normalized transcripts of genes surrounding peak 1 identifies that BP1026B_II1198 positively regulates BP1026B_II0600. (**c**) Normalized transcripts of genes around peak 2 identify that BP1026B_II1198 negatively regulates BP1026B_II1006. (**d**) Infection of RAW264.7 cells by transposon mutants of BP1026B_II0600 and BP1026B_II1006 compared to wildtype *Bp* 1026b showing various levels of intracellular replication defect. (**e**) Annotated function of BP1026B_II0600 and BP1026B_II1006 and corresponding intracellular replication when compared to wildtype *Bp* 1026b (%WT). (**f**) Model for the direct regulation network of BP1026B_II1198. Data in bar graphs represent means ± s.e.m and analyzed via unpaired t-test. P values presented are as follows: *p < 0.05.

BP1026B_II1198 regulates numerous pathways/genes previously identified to be involved in virulence and many potential pathways/genes that have unknown functions during pathogenesis (Fig. 4, Fig. S1, Fig. S2). To determine the direct regulation network of BP1026B_II1198, we used a ChIP-seq strategy as previously described^2,3^ but modified for *Burkholderia* species. All peaks identified were plotted by genome position on each *Bp* chromosome revealing two major peaks located on chromosome II (Fig. 5a,b). When the –log_10_ of the q-value was compared to the fold-enrichment of each peak, we see the same two peaks further isolated from the background (Fig. 5c). These two peaks suggest that BP1026B_II1198 binds to two intergenic regions between divergently transcribed genes (Fig. 6a). Peak 1 is a 227 bp sequence in the intergenic region between levanase, levansucrase precursor, BP1026B_II0599-0600, and a LacI family regulator protein, BP1026B_II0601 (Fig. 6a). Peak 2 is a 508 bp region between BP1026B_II1006, a DNA-binding protein, and BP1026B_II1007, an oxidoreductase (Fig. 6a). By comparing the sequences of each of these peaks using MEME^38^, a 12 bp conserved region was identified (Fig. 5d). The sequence, AAAAGTAXXTTA, has seven and three conserved base pairs separated by a two base pair spacer and is oriented in the same direction on both peaks suggesting that transcriptional control from each region will be in the same direction. Emphasizing this point, the RNA-seq data shows that BP1026B_II1198 regulates genes in the same orientation by positively regulating BP1026B_II0600 and negatively regulating BP1026B_II1006 while showing no transcriptional control of genes in the opposite orientation, BP1026B_II0601 and BP1026B_II1007 (Fig. 6b,c).

### Genes directly controlled by BP1026B_II1198 are important during intracellular infection

To investigate the roles BP1026B_II0600 and BP1026B_II1006 play during intracellular pathogenesis, a RAW264.7 macrophage intracellular replication assay was carried out. At 24 hpi a transposon mutant of BP1026B_II0600, a levansucrase precursor that is up-regulated by BP1026B_II1198, replicated intracellularly to 51.62% of wildtype *Bp* 1026b (Fig. 6d,e). This is a drastic decrease indicating that BP1026B_II0600 has a role during intracellular pathogenesis. While levansucrase is used as a counter-selection marker in the manipulation of the *Bp* genome, induction of BP1026B_II1198 in the presence of sucrose did not show an additional decrease in growth (Fig. S3c). On the other hand, a transposon mutant of BP1026B_II1006, a DNA-binding protein that is down-regulated by BP1026B_II1198, showed 82.89% wildtype intracellular replication at 24 hpi (Fig. 6d,e). Although it has a small decrease in the intracellular replication compared to wildtype, this decrease could be significant considering that BP1026B_II1006 is a DNA-binding protein and could have downstream transcriptional effects on other pathways like the ones identified by RNA-seq analysis. These data show that BP1026B_II1198 has a direct affect on the positive transcriptional control of BP1026B_II0600 and the negative transcriptional control of BP1026B_II1006 leading to variations in the pathogenesis during RAW264.7 murine macrophage infection (Fig. 6f).

## Discussion

Bacterial transcriptional networks are highly complex and dependent on many variables to properly function. When bacteria are exposed to a unique environment they sense and respond through signaling pathways that lead to the expression of proteins/factors aiding in survival. This is especially true during infection, as pathogenic bacteria encounter various immune defenses within the host. *Pseudomonas aeruginosa* (*Pa*) is a model organism for the study of biofilms and regulation of virulence factor production, which are controlled by a complex network of transcription factors and signaling systems^39^. Only recently are the subtleties of the *Pa* transcription factor regulation network coming to light, highlighted by significant functional crosstalk between transcription factors and regulation systems^40,41^. *Mycobacterium tuberculosis* also has a well-documented, highly complex, regulatory network that it uses to navigate hypoxic environments^42^. To fully understand the pathogenic processes that allow for *Bp* host colonization and infection we must understand how these pathways are regulated. Some studies have started to shed light on the intricacies of *Bp* virulence regulation^14,17,43,44^. In the present study, we add to the knowledge of the *Bp* regulatory network during intracellular infection through the identification and characterization of BP1026B_II1198.

BP1026B_II1198 is annotated as a PadR-like transcriptional regulator. PadR regulators were first identified in *Pediococcus pentosaceus* and later characterized to act as repressors of phenolic acid decarboxylase, encoded by the *padA* gene in *Lactobacillus plantarum*^45,46^. Other PadR-like regulators have been identified in other survival processes including drug resistance, stress responses, biosynthesis of antibiotics, and carbon catabolism^47–53^. The Pfam database indicates that there are over 19,000 sequences containing a PadR-like domain and over 2,900 sequences representing the domain architecture of BP1026B_II1198 with the N-terminal PadR domain and a C-terminal virulence activator alpha domain^19^. PadR domains have also been shown to be in combination with many other domains with a total of 35 domain architectures identified in the Pfam database^19^. *Bp* does not contain a *padA* homolog or a phenolic acid metabolic pathway, and the varied nature of PadR-like proteins suggests broad and diverse functionalities. We therefore took a broad approach to study BP1026B_II1198.

BP1026B_II1198 is differentially expressed during the *Bp* intracellular lifecycle and is required for in vitro and in vivo pathogenesis, highlighted by the 100% attenuation of the BP1026B_II1198 mutant in the BALB/c mouse model. These two observations show that *Bp* precisely controls pathogenic function within the host. To further understand this contribution to pathogenesis we identified the genes controlled by BP1026B_II1198 using RNA-seq. We observed that 79.8% of genes differentially regulated in the presence of BP1026B_II1198 have no known functions. This is a striking increase from the 26.7% of genes annotated as hypothetical unknowns within the *Bp* 1026b genome, suggesting that many pathogenic and survival pathways are yet to be discovered. The presence of BP1026B_II1198 during the later stages of infection coincides with the regional expression around the T6SS-5 locus, aligning with the current understanding of the *Bp* lifecycle. In addition, expression of BP1026B_II1198 leads to the up-regulation of several secondary metabolite gene clusters malleobactin^23^, pyochelin^24^, and malleilactone^25^, that have been previously identified to be involved in iron acquisition and pathogenesis. The regulation of these known virulence mechanisms likely contribute to the attenuation of the BP1026B_II1198 mutant, although we speculate that there are other factors that are also involved.

We probed many genes that were regulated by BP1026B_II1198 to identify contributors to pathogenesis, including genes that may have a less direct pathogenic function. Although Fe-S clusters were first identified in 1962 to be involved during electron transport^54^, their function is diverse and has been implicated in the modulation of gene expression^55^. Fe-S clusters can respond to increased oxidative stress, iron levels within the host, or other environmental signals during infection leading to an adaptive response^56^. The up-regulation of genes involved in Fe-S assembly (BP1026B_I0955, BP1026B_I0956, NifU, SufD) by BP1026B_II1198, could be induced in response to the intracellular environment, where iron-limitation, reactive-oxygen-species, and other environmental cues requires the sensitive nature of Fe-S regulatory complexes. This result correlates with previous reports showing up-regulation of Fe-S assembly genes in *Burkholderia mallei*, a clonal derivative of *Bp,* when exposed to sub-inhibitory levels of nitric oxide^57^. Genes involved in nitrogen metabolism were also up-regulated, showing that BP1026B_II1198 is partially responsible for a shift in general metabolism during infection. In addition to this, the BP1026B_I1020 mutant showed a reduction during intracellular replication in RAW264.7 cells.

Other mutants of genes regulated by BP1026B_II1198 that showed reductions in RAW264.7 pathogenesis have less clues as to their function. BP1026B_II1506 is a GntR family transcriptional regulator that could have many downstream gene expression changes that contribute to the reduction in virulence while BP1026B_II1581 is a hypothetical protein that is worth further investigation. BP1026B_I1775 is a Sec-secreted lipoprotein with cleavage site located between amino acids 28 and 29 as identified by SignalP5.0 analysis^58^. The function of this protein should be investigated further and could potentially be manipulated as a novel therapeutic target or subunit vaccine component. BP1026B_II1736 sits within the second type III secretion system (T3SS-2) in *Bp* and encodes SctK, a cytoplasmic structural component of the type III injectasome^59^. This protein along with SctQ and SctL are referred to as the ‘sorting platform’ and play a critical role on the order in which effector proteins are secreted^60^. Beyond the implications the specific potential function of BP1026B_II1736, it has been shown that T3SS-2 is involved in the pathogenesis of *Solanum lycopersicum* (tomato) but not mammalian cells^37^. While T3SS-2 is not singly required for complete pathogenesis in mammalian models of infection^61^, our data suggest that this system could contribute to pathogenesis.

Large numbers of genes/pathways are controlled indirectly through the expression of BP1026B_II1198 as described above. To find the direct DNA binding regions of BP1026B_II1198 we used a ChIP-seq strategy. Through this approach we identified two major regions of DNA binding in intergenic regions of divergently transcribed genes. BP1026B_II1198 showed up-regulation of BP1026B_II0600 and down regulation of BP1026B_II1006. This suggests that BP1026B_II1198 has a dual function as an activator and as a repressor, similar to other PadR-type regulators previously described^50^. Subsequent mutant analysis of these genes showed a 52% and 83% reduction in wildtype intracellular replication. BP1026B_II0600 encodes a levansucrase precursor, which to our knowledge, has yet to be described as a virulence factor during infection of mammalian cells. However, in plant pathogens like *Pseudomonas syringae*, levansucrase is critical for the formation of levans that contribute to exopolysaccaride production^62,63^. Because *Bp* is an incidental host of mammals, and freely lives within the environment, the expression of levansucrase could indicate that this gene is used for adaptation to multiple conditions. BP1026B_II1006 is annotated as a DNA binding protein but further analysis using Phyre2^64^ predicts structural similarities to an Fe-S complex containing transcriptional regulator, RrsR^65^. We predict that the down-regulation of BP1026B_II1006 by BP1026B_II1198 plays a major role in the indirect gene expression changes that occur in our data set. Although outside of the scope of the present work, this data warrants further analysis of the regulation network of BP1026B_II1006.

Here we show a snapshot of what is going on during intracellular infection through the lens of the transcriptional regulator BP1026B_II1198 that we would like to tentatively name Intracellular Pathogenesis Regulator A (IprA). The loss of IprA results in significantly reduced pathogenesis in RAW264.7 murine macrophages and in BALB/c mice. This investigation identified that IprA functions as a transcriptional regulator during *Bp* intracellular transit through the host by activation/repression of multiple factors and pathways. Although, no single virulence factor controlled by IprA shows the same drastic decrease in pathogenesis we believe the cumulative effect of the changes in transcription by IprA highlights its importance during pathogenesis. We believe that in the intracellular environment, many transcription factors, including IprA, work in unison to control how *Bp* responds to specific environments, allowing for infection to occur. The future study of many of these transcriptional regulators (i.e. BP1026B_II1006 and BP1026B_II1506) will increase the resolution of what we know about the *Bp* intracellular lifecycle and *Bp* gene expression within the host. Although the data represent the expression profile of one transcription factor, it is one step towards a better understanding of the intricacies of *Bp* intracellular pathogenesis.

## Methods and materials

### Bacterial strains, media and culture conditions

*Escherichia coli* strain EPMax10B (BioRad), E1869, and E1354 were used for cloning or plasmid mobilization into *Bp* as described previously^66,67^. *Bp* 1026b and its select agent regulation excluded analogue, *Bp*82, were used as described in specific sections below. Luria–Bertani (LB) medium (Difco) or 1 × M9 minimal medium supplemented with 20 mM glucose (MG) were used to culture all strains. MG media was supplemented with 0.3% glyphosate (GS) or 0.1% chlorinated phenylalanine (cPhe) when appropriate. Growth media for *Bp*82 was supplemented with adenine and thiamine as previously described^68^. Experiments involving the manipulation of *Bp* were conducted in a CDC-approved and registered facility at the University of Hawaiʻi at Mānoa or Colorado State University. All work with the select agent *Bp* was approved by internal review and adhere to recommendations set forth in the BMBL, 5^th^ edition^69^ for BSL3 organisms.

### Transposon mutants tested as potential virulence factors

The sequence-defined transposon mutant library of *Bp* 1026b was used as a source of mutants to test for potential virulence factors^36^. Mutants of BP1026B_I0528, BP1026B_I0950, and BP1026B_II1464 were not tested because the library did not have insertions in any of these genes^36^. Transposon mutants available in the transposon library were struck for isolation on media containing kanamycin and each insertion site sequenced to confirm correct transposon insertion location (data not shown). The transposon insertion in BP1026B_II1769 was not validated and confirmed to be present in another gene, BP1026B_II2115, a putative diguanylate cyclase. BP1026B_II1769 lacked a second transposon insertion. All other transposon mutants tested were confirmed to be in the gene of interest by sequencing.

### Molecular methods and reagents

Molecular methods and reagents were used as described previously^20,67,70,71^. An in-frame deletion mutant of BP1026B_II1198 was generated in *Bp* 1026b using lambda-red recombineering with minor modification^72^. Lambda red genes were PCR amplified from pKaKa1 and co-incubated with the knockout fragment of BP1026B_II1198 and selection carried out on glyphosate. Glyphosate resistant colonies were purified and patched on MG + 0.3% GS and screened for presence of the *gat* gene to confirm the deletion BP1026B_II1198. The *gat-pheS* cassette was removed via Flp recombination and growth on MG + 0.1% cPhe. Phenotypes were confirmed after purification and patching on corresponding media (Fig. S4a). For expression of BP1026B_II1198 (complementation, RNA-seq, ChIP-seq), BP1026B_II1198 was PCR amplified from *Bp* 1026b with oligos 2778 (5’-GGT TGC CTC GCA TAT GTC CCT GCC CCA C) and 2779 (5’-TCG AAG CTT CAA GCC GAC AAC GAT CTT) digested with *Nde*I and *Hin*dIII and ligated into *Nde*I/*Hin*dIII-cut pAM3GIQ-3xTY1 (Fig. S4b). Confirmation of the resulting vector, pAM3GIQ-3xTY1-BP1026B_II1198, was carried out by *Eco*RV/*Phs*AI and *Nde*I/*Hin*dIII digests and the expression of BP1026B_II1198 confirmed via western blot analysis with a mouse anti-TY1-tag monoclonal antibody (Diagenode C15200054) and goat anti-mouse conjugated to HRP (Invitrogen G-21040) (Fig. S4c).

### Growth analysis

Growth analysis was carried out as previously described with minor modifications^68^. All strains were first grown overnight in LB broth at 37 °C, harvested the following day, diluted to an OD_630_ of 0.1 in 200 µl of fresh LB, and placed into 96 well plate. Growth curves were carried out at 37 °C shaking and data was recorded with the BioTek ELx808IU plate reader, measurements taken at OD_630_ every 30 min.

### Cell infection assays

Intracellular replication and MNGC formation assays were carried out as previously described^73^ with minor modifications. Briefly, RAW264.7 murine macrophages were seeded for infection at 80–90% confluence on Corning CellBIND culture plates, allowed to attach overnight, washed twice with 1XPBS, and infected the following day. For intracellular replication assays, *Bp* 1026b, the BP1026B_II1198 mutant, or the BP1026B_II1198 complement (mutant with pAM3GIQ-3xTY1-BP1026B_II1198) were allowed to infect monolayers at an MOI of 1:1, washed with 1XPBS, and then DMEM supplemented with 10% FBS, 0.1 mM IPTG to drive expression of the complement, 700 µg/mL amikacin and 700 µg/mL kanamycin were added to kill any extracellular bacteria. At 2, 8, and 24 hpi, infected monolayers were lysed with 0.2% Triton X-100. Serial dilutions of lysates were plated on LB and colony-forming units (CFU) per well were determined. MNGC formation assays were carried out as intracellular replication assays, with the exception that 1.2% low-melt agarose was added to DMEM after infection allowing the formation of MNGCs. At 24 h post infection, monolayers were fixed with 4% paraformaldehyde, agarose plugs removed, stained with 0.05% crystal violet, and MNGC diameters measured with the Zeiss Axio Observer D1 and the AxioVision 64 bit 4.9.1 software. Cell infection assays with mutants from the *Bp* 1026b::T24 transposon mutant library were carried out as described above although kanamycin was removed and 1,500 µg/mL amikacin added to remove extracellular bacteria post infection. Monolayers were lysed at 24 h post infection as described above. Percent wildtype replication (%WT) was calculated by the following formula: %WT = (CFU_mutant_/mean CFU_WT_) × 100.

### Animal studies

BALB/c mice (4 to 6 weeks old) were purchased from Charles River Laboratory, allowed to acclimatize for a week, and then infected with *Bp* 1026b and the BP1026B_II1198 mutant as previously described^7^. Each infection group had 5 mice and the inoculating dose was 4,500 CFU via the intranasal route after mice were anesthetized with ketamine (100 mg/kg) and xylazine (10 mg/kg). Mice were monitored for 62 days post infection and organs (lungs, liver, and spleen) from surviving mice were harvested, homogenized in PBS, serial diluted, and plated on LB. The only surviving mice that survived the length of the study were mice infected with the BP1026B_II1198 mutant. The organ loads show the persistence of this mutant within the BALB/c mouse. No mice were excluded from the study or analysis. Survival curves were plotted using Prism software and analyzed by Log-rank (Mantel-Cox) test (GraphPad).

### RNA-seq, RT-qPCR, and ChIP-seq analysis

RNA-seq and ChIP-seq analysis were carried out under the same conditions. Briefly, *Bp*82 expressing BP1026B_II1198 from pAM3GIQ-3xTY1-BP1026B_II1198 was grown overnight and sub-cultured to mid-log phase in LB + adenine + 0.1 mM IPTG in triplicate. An empty vector (pAM3GIQ-3xTY1) was used as a control. Total RNA was harvested using RNeasy Mini Kit (Qiagen) with on-column (Qiagen) and off-column (Epicentere) DNase digestion steps. RNA samples were sent to Tufts University Genomics Core (TUCF Genomics) for library preparation and Illumina 50 bp single-end reads were sequenced on the Illumina HiSeq 2500. RNA-seq data was analyzed with Rockhopper^21^ to identify individual genes controlled by BP1026B_II1198 and WoPPER^22^ to determine larger regions of the genome that are controlled by BP1026B_II1198. RNA-seq data was validated by RT-qPCR using BP1026B_I0774, BP1026B_II1384, and BP1026B_II0521 as housekeeping genes due to consistent expression (log_2_FC = 0) between conditions (Fig. S4d). Primers (Table S2) were designed with Integrated DNA Technologies Primer Quest software. Reverse transcription was carried out with Superscript III (Invitrogen) and Power Up SYBR Green master mix (Applied Biosystems) was used for amplification and quantitation on an Eppendorf Realplex 4 thermocycler. RT-qPCR data was analyzed as previously described^7,74^.

ChIP-seq was carried out as previously described with minor modifications^2,3^. Briefly, ChIP-seq samples were grown identically as RNA-seq samples, harvested, and fixed with 4% paraformaldehyde, followed by shearing of DNA–protein complexes with the Covaris M220 ultrafocused sonicator. Cell debris was removed and DNA–protein complexes were immunoprecipitated with anti-TY1-tag monoclonal antibody (Diagenode C15200054) and secondary antibodies conjugated to magnetic beads (Diagenode C03010022). DNA–protein complexes were washed, decrosslinked, treated with RNaseA and proteinase K, and purified with QIAquick PCR purification kit. Immunoprecipitated DNA was sent to TUCF Genomics where DNA libraries were prepped and 50 bp single-end reads were sequenced with the Illumina HiSeq 2500. ChIP-seq data was aligned to the *Bp* 1026b genome with Bowtie2^75^, peaks called with MACS2^76^, and consensus binding regions determined with MEME^38^.

### Ethical standards

All animal studies described in this manuscript were approved by the Institutional Animal Care and Use Committee at the University of Hawaiʻi at Mānoa (Protocol No. 10-1073), conducted in compliance with the NIH (National Institutes of Health) Guide for the Care and Use of Laboratory Animals, and in compliance with ARRIVE guidelines.

## Supplementary Information

Supplementary Information 1.

Supplementary Information 2.

Supplementary Information 3.

## Data Availability

The datasets and materials generated during the current study are available from the corresponding author upon reasonable request. Any transfer of select agent materials needs additional approval from Bradley R. Borlee and the Responsible Official at Colorado State University. Any select agent transfer must be to a select agent registered facility, approved by the CDC, and comply with all select agent regulations (selectagents.gov).
