# Supplementary material for: Identification of a PadR-type regulator essential for intracellular pathogenesis of Burkholderia pseudomallei
Source: Sci Rep. 2021 May 17;11:10405. doi: 10.1038/s41598-021-89852-7 (PMC8128862; doi:10.1038/s41598-021-89852-7)
Supplement: Supplementary file 1 — Supplementary Information 1. [file 41598_2021_89852_MOESM1_ESM.pdf]

Supplemental information for:

Identification of a PadR-Type Regulator essential for Intracellular Pathogenesis of *Burkholderia pseudomallei*

Ian A. McMillan[1], Michael H. Norris[1,2], Jan Zarzycki-Siek[1], Yun Heacock-Kang[1], Zhenxin Sun[1], Bradley R. Borlee[3], and Tung T. Hoang[1*]

[1]School of Life Sciences, University of Hawai‘i at Mānoa, Honolulu, Hawai‘i, United States of America; [2]Present address: Department of Geography and Emerging Pathogens Institute, University of Florida, Gainesville, Florida, United States of America; [3]Department of Microbiology, Immunology, and Pathology, Colorado State University, Fort Collins, Colorado, United States of America

Running title: *Bp* pathogenic regulation by BP1026B_II1198

* Address correspondence to Tung T. Hoang, Tel: +1 808 956 3522; Fax: +1 808 956 5339; e-mail: tongh@hawaii.edu

a

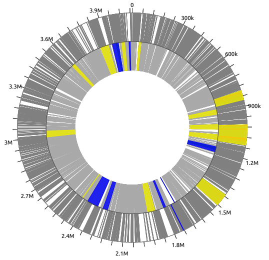

b

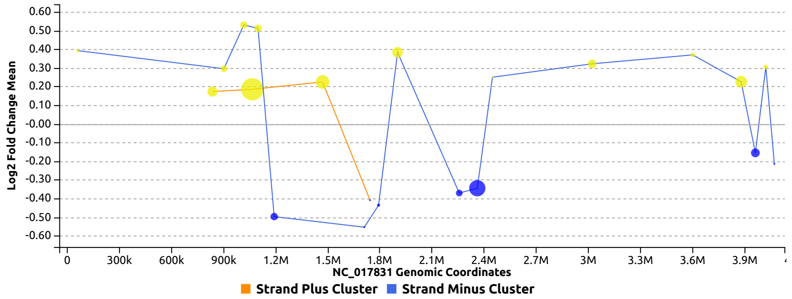

c

Cluster	Start Coordinates	End Coordinates	Strand	Gene ID	Expression
1	50668	66682	-	BP1026B_I0048;BP1026B_I0049;BP1026B_I0050;BP1026B_I0051;BP1026B_I0052;BP1026B_I0053;BP1026B_I0054;BP1026B_I0055;BP1026B_I0056;BP1026B_I0057;BP1026B_I0060;BP1026B_I0061;BP1026B_I0062	UP
2	809024	862500	+	BP1026B_I0767;BP1026B_I0769;BP1026B_I0777;BP1026B_I0778;BP1026B_I0780;BP1026B_I0785;BP1026B_I0786;BP1026B_I0792;BP1026B_I0793;BP1026B_I0794;BP1026B_I0795;BP1026B_I0796;BP1026B_I0797;BP1026B_I0798;BP1026B_I0799;BP1026B_I0800;BP1026B_I0801;BP1026B_I0802;BP1026B_I0803;BP1026B_I0804;BP1026B_I0805;BP1026B_I0806;BP1026B_I0808	UP
3	885997	921194	-	BP1026B_I0831;BP1026B_I0832;BP1026B_I0834;BP1026B_I0835;BP1026B_I0836;BP1026B_I0837;BP1026B_I0840;BP1026B_I0841;BP1026B_I0844;BP1026B_I0845;BP1026B_I0846;BP1026B_I0848;BP1026B_I0849;BP1026B_I0852;BP1026B_I0858;BP1026B_I0859;BP1026B_I0860;BP1026B_I0861;BP1026B_I0862;BP1026B_I0863	UP
4	998745	1036089	-	BP1026B_I0940;BP1026B_I0942;BP1026B_I0943;BP1026B_I0944;BP1026B_I0945;BP1026B_I0946;BP1026B_I0947;BP1026B_I0949;BP1026B_I0951;BP1026B_I0952;BP1026B_I0953;BP1026B_I0954;BP1026B_I0955;BP1026B_I0956;BP1026B_I0957;BP1026B_I0958;BP1026B_I0962;BP1026B_I0968;BP1026B_I0969;BP1026B_I0970;BP1026B_I0971;BP1026B_I0972;BP1026B_I0973;BP1026B_I0974;BP1026B_I0975	UP
5	1006199	1122917	+	BP1026B_I0948;BP1026B_I0950;BP1026B_I0959;BP1026B_I0960;BP1026B_I0961;BP1026B_I0963;BP1026B_I0964;BP1026B_I0965;BP1026B_I0966;BP1026B_I0967;BP1026B_I0976;BP1026B_I0977;BP1026B_I0978;BP1026B_I0980;BP1026B_I0979;BP1026B_I0981;BP1026B_I0982;BP1026B_I0983;BP1026B_I0984;BP1026B_I0985;BP1026B_I0986;BP1026B_I0987;BP1026B_I0988;BP1026B_I0989;BP1026B_I0990;BP1026B_I0991;BP1026B_I0992;BP1026B_I0993;BP1026B_I0994;BP1026B_I0995;BP1026B_I0997;BP1026B_I0999;BP1026B_I1000;BP1026B_I1003;BP1026B_I1004;BP1026B_I1005;BP1026B_I1006;BP1026B_I1007;BP1026B_I1009;BP1026B_I1010;BP1026B_I1011;BP1026B_I1023;BP1026B_I1028;BP1026B_I1029;BP1026B_I1030;BP1026B_I1033;BP1026B_I1034;BP1026B_I1036;BP1026B_I1037;BP1026B_I1038;BP1026B_I1039;BP1026B_I1040;BP1026B_I1041;BP1026B_I1042;BP1026B_I1043;BP1026B_I1044	UP
6	1076351	1115883	-	BP1026B_I1008;BP1026B_I1012;BP1026B_I1013;BP1026B_I1014;BP1026B_I1015;BP1026B_I1016;BP1026B_I1017;BP1026B_I1018;BP1026B_I1019;BP1026B_I1020;BP1026B_I1021;BP1026B_I1024;BP1026B_I1025;BP1026B_I1026;BP1026B_I1027;BP1026B_I1031;BP1026B_I1032;BP1026B_I1035	UP
7	1169046	1208323	-	BP1026B_I1089;BP1026B_I1125;BP1026B_I1126;BP1026B_I1127;BP1026B_I1128;BP1026B_I1129;BP1026B_I1130;BP1026B_I1131;BP1026B_I1139	DOWN
8	1431508	1503022	+	BP1026B_I1329;BP1026B_I1332;BP1026B_I1333;BP1026B_I1334;BP1026B_I1335;BP1026B_I1336;BP1026B_I1339;BP1026B_I1340;BP1026B_I1342;BP1026B_I1344;BP1026B_I1347;BP1026B_I1351;BP1026B_I1352;BP1026B_I1358;BP1026B_I1359;BP1026B_I1360;BP1026B_I1361;BP1026B_I1363;BP1026B_I1364;BP1026B_I1365;BP1026B_I1366;BP1026B_I1367;BP1026B_I1370;BP1026B_I1371;BP1026B_I1372;BP1026B_I1373;BP1026B_I1374;BP1026B_I1375;BP1026B_I1376;BP1026B_I1377;BP1026B_I1378;BP1026B_I1379;BP1026B_I1383;BP1026B_I1388;BP1026B_I1391;BP1026B_I1392;BP1026B_I1393;BP1026B_I1394;BP1026B_I1395;BP1026B_I1396;BP1026B_I1397;BP1026B_I1398	UP
9	1711272	1712173	-	BP1026B_I1582;BP1026B_I1584	DOWN
10	1742675	1744982	+	BP1026B_I1607;BP1026B_I1608;BP1026B_I1609	DOWN
11	1786537	1802986	-	BP1026B_I1649;BP1026B_I1650;BP1026B_I1651;BP1026B_I1658;BP1026B_I1663	DOWN
12	1872678	1928301	-	BP1026B_I1708;BP1026B_I1711;BP1026B_I1712;BP1026B_I1713;BP1026B_I1715;BP1026B_I1722;BP1026B_I1723;BP1026B_I1724;BP1026B_I1725;BP1026B_I1726;BP1026B_I1727;BP1026B_I1731;BP1026B_I1732;BP1026B_I1733;BP1026B_I1734;BP1026B_I1735;BP1026B_I1738;BP1026B_I1739;BP1026B_I1740	UP
13	2237014	2272171	-	BP1026B_I2004;BP1026B_I2005;BP1026B_I2006;BP1026B_I2008;BP1026B_I2009;BP1026B_I2011;BP1026B_I2013;BP1026B_I2014;BP1026B_I2015;BP1026B_I2016;BP1026B_I2017;BP1026B_I2018;BP1026B_I2019;BP1026B_I2020;BP1026B_I2021;BP1026B_I2022;BP1026B_I2023;BP1026B_I2024;BP1026B_I2025;BP1026B_I2026;BP1026B_I2027;BP1026B_I2028;BP1026B_I2029;BP1026B_I2030;BP1026B_I2031;BP1026B_I2034	DOWN
14	2315668	2401162	-	BP1026B_I2063;BP1026B_I2064;BP1026B_I2066;BP1026B_I2074;BP1026B_I2080;BP1026B_I2082;BP1026B_I2083;BP1026B_I2085;BP1026B_I2087;BP1026B_I2088;BP1026B_I2089;BP1026B_I2090;BP1026B_I2091;BP1026B_I2092;BP1026B_I2093;BP1026B_I2094;BP1026B_I2095;BP1026B_I2096;BP1026B_I2097;BP1026B_I2098;BP1026B_I2099;BP1026B_I2100;BP1026B_I2101;BP1026B_I2102;BP1026B_I2103;BP1026B_I2104;BP1026B_I2105;BP1026B_I2106;BP1026B_I2107;BP1026B_I2108;BP1026B_I2109;BP1026B_I2110;BP1026B_I2111;BP1026B_I2112;BP1026B_I2113;BP1026B_I2114;BP1026B_I2115;BP1026B_I2116;BP1026B_I2119;BP1026B_I2120;BP1026B_I2121;BP1026B_I2122;BP1026B_I2124;BP1026B_I2125;BP1026B_I2126;BP1026B_I2127;BP1026B_I2128;BP1026B_I2129;BP1026B_I2130;BP1026B_I2131;BP1026B_I2132;BP1026B_I2134;BP1026B_I2135;BP1026B_I2137;BP1026B_I2138;BP1026B_I2140;BP1026B_I2144;BP1026B_I2145;BP1026B_I2157;BP1026B_I2158;BP1026B_I2159;BP1026B_I2167;BP1026B_I2168;BP1026B_I2170;BP1026B_I2172;BP1026B_I2173;BP1026B_I2174	DOWN
15	2445406	2447447	-	BP1026B_I2215;BP1026B_I2217	UP
16	2997776	3043477	-	BP1026B_I2703;BP1026B_I2704;BP1026B_I2706;BP1026B_I2707;BP1026B_I2708;BP1026B_I2713;BP1026B_I2715;BP1026B_I2718;BP1026B_I2719;BP1026B_I2720;BP1026B_I2721;BP1026B_I2722;BP1026B_I2723;BP1026B_I2724;BP1026B_I2725;BP1026B_I2730;BP1026B_I2731;BP1026B_I2733;BP1026B_I2735;BP1026B_I2736;BP1026B_I2737;BP1026B_I2739;BP1026B_I2740;BP1026B_I2741;BP1026B_I2742	UP
17	3589655	3612278	-	BP1026B_I3213;BP1026B_I3214;BP1026B_I3215;BP1026B_I3223;BP1026B_I3224;BP1026B_I3225;BP1026B_I3227;BP1026B_I3228;BP1026B_I3230;BP1026B_I3231;BP1026B_I3232;BP1026B_I3233;BP1026B_I3234	UP
18	3846410	3907946	-	BP1026B_I3480;BP1026B_I3482;BP1026B_I3484;BP1026B_I3489;BP1026B_I3494;BP1026B_I3496;BP1026B_I3497;BP1026B_I3499;BP1026B_I3501;BP1026B_I3504;BP1026B_I3505;BP1026B_I3506;BP1026B_I3507;BP1026B_I3509;BP1026B_I3513;BP1026B_I3515;BP1026B_I3517;BP1026B_I3519;BP1026B_I3520;BP1026B_I3523;BP1026B_I3524;BP1026B_I3525;BP1026B_I3526;BP1026B_I3527;BP1026B_I3528;BP1026B_I3529;BP1026B_I3530;BP1026B_I3531;BP1026B_I3532;BP1026B_I3533;BP1026B_I3534;BP1026B_I3535;BP1026B_I3536;BP1026B_I3537	UP
19	3940194	3986387	-	BP1026B_I3569;BP1026B_I3570;BP1026B_I3571;BP1026B_I3572;BP1026B_I3575;BP1026B_I3577;BP1026B_I3578;BP1026B_I3580;BP1026B_I3582;BP1026B_I3583;BP1026B_I3584;BP1026B_I3585;BP1026B_I3592;BP1026B_I3593;BP1026B_I3622;BP1026B_I3623;BP1026B_I3624	DOWN
20	4010941	4034609	-	BP1026B_I3653;BP1026B_I3656;BP1026B_I3658;BP1026B_I3659;BP1026B_I3660;BP1026B_I3661;BP1026B_I3664;BP1026B_I3665;BP1026B_I3666;BP1026B_I3669;BP1026B_I3670;BP1026B_I3671;BP1026B_I3672;BP1026B_I3673	UP
21	4067419	4077202	-	BP1026B_I3701;BP1026B_I3702;BP1026B_I3703;BP1026B_I3704;BP1026B_I3705;BP1026B_I3706;BP1026B_I3707;BP1026B_I3708;BP1026B_I3709	DOWN

Supplemental Figure 1| WoPPER analysis reveals 21 gene clusters regulated by BP1026B_II1198 on *Bp* 1026b chromosome 1. a) Circular schematic of chromosome 1 showing up (yellow) and down (blue) regulated clusters of genes. b) Linear plot showing gene clusters that are regulated by BP1026B_II1198 and the mean of the clusters log_2 fold change. Size of the circle represents the number of genes in each cluster; yellow is up-regulated and blue is down-regulated. c) Table listing each cluster of genes.

a

b

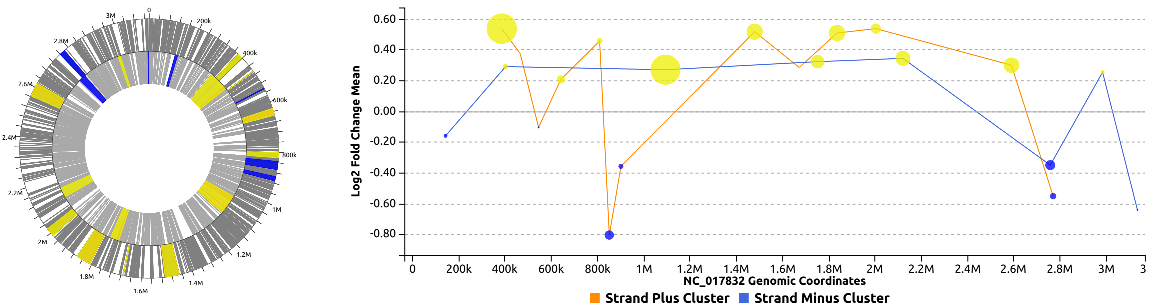

c

Cluster	Start Coordinates	End Coordinates	Strand	Gene ID	Expression
1	135381	148579	-	BP1026B_II0116;BP1026B_II0117;BP1026B_II0118;BP1026B_II0119;BP1026B_II0120;BP1026B_II0121;BP1026B_II0122;BP1026B_II0124;BP1026B_II0126	DOWN
2	350868	455171	-	BP1026B_II0291;BP1026B_II0292;BP1026B_II0294;BP1026B_II0296;BP1026B_II0298;BP1026B_II0300;BP1026B_II0301;BP1026B_II0304;BP1026B_II0305;BP1026B_II0306;BP1026B_II0307;BP1026B_II0308;BP1026B_II0310;BP1026B_II0311;BP1026B_II0312;BP1026B_II0314;BP1026B_II0318;BP1026B_II0319;BP1026B_II0324;BP1026B_II0325;BP1026B_II0327;BP1026B_II0328;BP1026B_II0329;BP1026B_II0330;BP1026B_II0331;BP1026B_II0332;BP1026B_II0333;BP1026B_II0334;BP1026B_II0335;BP1026B_II0336;BP1026B_II0337;BP1026B_II0338;BP1026B_II0339;BP1026B_II0340;BP1026B_II0348;BP1026B_II0349;BP1026B_II0350;BP1026B_II0356	UP
3	377577	395968	+	BP1026B_II0309;BP1026B_II0313;BP1026B_II0315;BP1026B_II0316;BP1026B_II0317;BP1026B_II0320;BP1026B_II0321;BP1026B_II0322;BP1026B_II0323	UP
4	464366	466075	+	BP1026B_II0362;BP1026B_II0363;BP1026B_II0364	UP
5	542392	546021	+	BP1026B_II0439;BP1026B_II0441;BP1026B_II0442;BP1026B_II0445	DOWN
6	627250	655441	+	BP1026B_II0509;BP1026B_II0510;BP1026B_II0511;BP1026B_II0512;BP1026B_II0513;BP1026B_II0514;BP1026B_II0525;BP1026B_II0526;BP1026B_II0527;BP1026B_II0528;BP1026B_II0529;BP1026B_II0530;BP1026B_II0531;BP1026B_II0533	UP
7	800331	820894	+	BP1026B_II0645;BP1026B_II0646;BP1026B_II0647;BP1026B_II0648;BP1026B_II0649;BP1026B_II0650;BP1026B_II0651;BP1026B_II0652;BP1026B_II0653;BP1026B_II0654	UP
8	834697	866695	+	BP1026B_II0670;BP1026B_II0681;BP1026B_II0683;BP1026B_II0682;BP1026B_II0684;BP1026B_II0685;BP1026B_II0686;BP1026B_II0690;BP1026B_II0691;BP1026B_II0694;BP1026B_II0695;BP1026B_II0696	DOWN
9	893813	911114	+	BP1026B_II0723;BP1026B_II0725;BP1026B_II0726;BP1026B_II0729;BP1026B_II0730;BP1026B_II0731;BP1026B_II0732;BP1026B_II0733;BP1026B_II0734;BP1026B_II0736;BP1026B_II0738;BP1026B_II0740;BP1026B_II0741	DOWN
10	1042707	1145759	-	BP1026B_II0850;BP1026B_II0854;BP1026B_II0863;BP1026B_II0875;BP1026B_II0876;BP1026B_II0877;BP1026B_II0878;BP1026B_II0879;BP1026B_II0880;BP1026B_II0884;BP1026B_II0885;BP1026B_II0886;BP1026B_II0891;BP1026B_II0892;BP1026B_II0898;BP1026B_II0899;BP1026B_II0907;BP1026B_II0911;BP1026B_II0912;BP1026B_II0913;BP1026B_II0916;BP1026B_II0918;BP1026B_II0920;BP1026B_II0921;BP1026B_II0922;BP1026B_II0930;BP1026B_II0932;BP1026B_II0933;BP1026B_II0934	UP
11	1452134	1507803	+	BP1026B_II1162;BP1026B_II1172;BP1026B_II1173;BP1026B_II1175;BP1026B_II1176;BP1026B_II1179;BP1026B_II1182;BP1026B_II1183;BP1026B_II1184;BP1026B_II1185;BP1026B_II1186;BP1026B_II1187;BP1026B_II1188;BP1026B_II1189;BP1026B_II1190;BP1026B_II1191;BP1026B_II1192;BP1026B_II1193;BP1026B_II1194;BP1026B_II1198;BP1026B_II1199;BP1026B_II1200;BP1026B_II1201;BP1026B_II1204;BP1026B_II1212	UP
12	1669880	1672481	+	BP1026B_II1329;BP1026B_II1332	UP
13	1728917	1775252	-	BP1026B_II1367;BP1026B_II1368;BP1026B_II1370;BP1026B_II1377;BP1026B_II1379;BP1026B_II1380;BP1026B_II1382;BP1026B_II1385;BP1026B_II1386;BP1026B_II1387;BP1026B_II1388;BP1026B_II1389;BP1026B_II1390;BP1026B_II1391;BP1026B_II1392;BP1026B_II1394;BP1026B_II1398;BP1026B_II1399;BP1026B_II1400	UP
14	1808195	1864242	+	BP1026B_II1431;BP1026B_II1438;BP1026B_II1439;BP1026B_II1440;BP1026B_II1441;BP1026B_II1442;BP1026B_II1449;BP1026B_II1452;BP1026B_II1460;BP1026B_II1464;BP1026B_II1469;BP1026B_II1470;BP1026B_II1472;BP1026B_II1471;BP1026B_II1473;BP1026B_II1476	UP
15	1985961	2020894	+	BP1026B_II1567;BP1026B_II1568;BP1026B_II1569;BP1026B_II1571;BP1026B_II1572;BP1026B_II1574;BP1026B_II1576;BP1026B_II1577;BP1026B_II1578;BP1026B_II1580;BP1026B_II1581;BP1026B_II1579;BP1026B_II1582;BP1026B_II1583;BP1026B_II1584;BP1026B_II1585;BP1026B_II1590;BP1026B_II1588;BP1026B_II1591;BP1026B_II1592;BP1026B_II1594;BP1026B_II1593;BP1026B_II1595;BP1026B_II1596	UP
16	2092808	2144294	-	BP1026B_II1663;BP1026B_II1670;BP1026B_II1671;BP1026B_II1674;BP1026B_II1677;BP1026B_II1678;BP1026B_II1679;BP1026B_II1681;BP1026B_II1682;BP1026B_II1683;BP1026B_II1684;BP1026B_II1685;BP1026B_II1686;BP1026B_II1687;BP1026B_II1688;BP1026B_II1689;BP1026B_II1693;BP1026B_II1694;BP1026B_II1699;BP1026B_II1703;BP1026B_II1704;BP1026B_II1705;BP1026B_II1706;BP1026B_II1707;BP1026B_II1708;BP1026B_II1709;BP1026B_II1710	UP
17	2563238	2616561	+	BP1026B_II2052;BP1026B_II2054;BP1026B_II2056;BP1026B_II2060;BP1026B_II2062;BP1026B_II2063;BP1026B_II2068;BP1026B_II2076;BP1026B_II2077;BP1026B_II2078;BP1026B_II2079;BP1026B_II2080;BP1026B_II2081;BP1026B_II2095;BP1026B_II2096;BP1026B_II2097;BP1026B_II2098;BP1026B_II2101	UP
18	2741722	2776012	-	BP1026B_II2207;BP1026B_II2209;BP1026B_II2210;BP1026B_II2211;BP1026B_II2213;BP1026B_II2214;BP1026B_II2217;BP1026B_II2218;BP1026B_II2220;BP1026B_II2222;BP1026B_II2223;BP1026B_II2224;BP1026B_II2225;BP1026B_II2226;BP1026B_II2227;BP1026B_II2229;BP1026B_II2230;BP1026B_II2231;BP1026B_II2232	DOWN
19	2760314	2782139	+	BP1026B_II2216;BP1026B_II2221;BP1026B_II2228;BP1026B_II2233;BP1026B_II2235;BP1026B_II2236;BP1026B_II2238;BP1026B_II2239	DOWN
20	2974712	2988613	-	BP1026B_II2408;BP1026B_II2409;BP1026B_II2410;BP1026B_II2411;BP1026B_II2412;BP1026B_II2413;BP1026B_II2414;BP1026B_II2415;BP1026B_II2416;BP1026B_II2419;BP1026B_II2422	UP
21	3131658	3136611	-	BP1026B_II2531;BP1026B_II2535;BP1026B_II2536	DOWN

Supplemental Figure 2| WoPPER analysis reveals 21 gene clusters regulated by BP1026B_II1198 on *Bp* 1026b chromosome 2. a) Circular schematic of chromosome 2 showing up (yellow) and down (blue) regulated clusters of genes. b) Linear plot showing gene clusters that are regulated by BP1026B_II1198 and the mean of the clusters log_2 fold change. Size of the circle represents the number of genes in each cluster; yellow is up-regulated and blue is down-regulated. c) Table listing each cluster of genes.

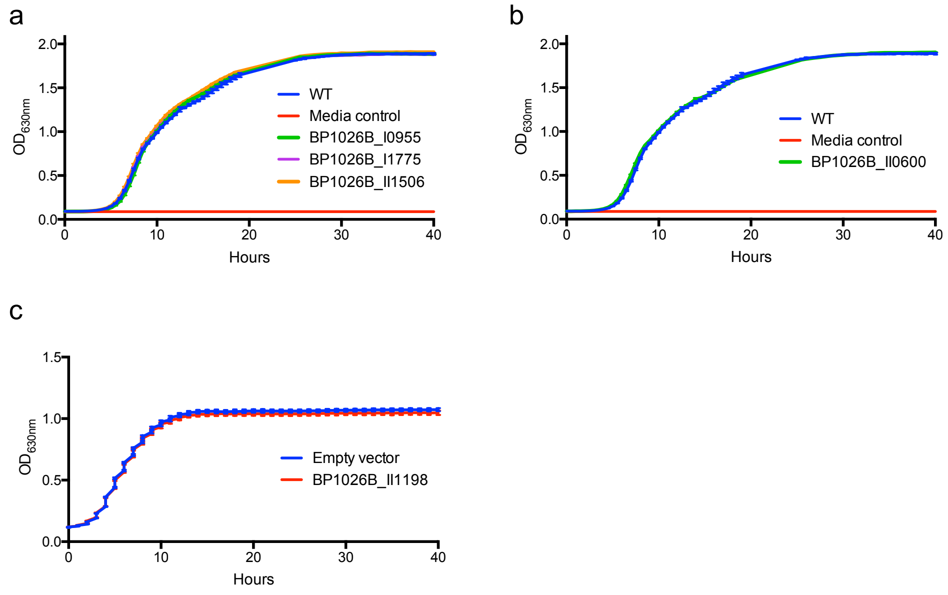

Supplemental Figure 3| Transposon mutants grow identical to wildtype *Bp* 1026b *in vitro* and induction of BP1026B_II1198 does not reduce growth in the presence of sucrose. a) Mutants controlled by BP1026B_II1198 indirectly (identified by RNA-seq) show no growth defects when grown in LB broth compared to wildtype *Bp* 1026b (WT). b) A BP1026B_II0600 mutant that is directly controlled by BP1026B_II1198 shows no defect when grown in LB broth compared to WT. c) IPTG induction of BP1026B_II1198 from pAM3GIQ-3xTY1 does not reduce growth in the presence of 10% sucrose compared to the empty vector control. Growth analysis was done in duplicate and the lines represent means ± s.e.m.

a

Strain	Phenotype
Bp 1026b	GS_S $cPhe_R$
BP1026B_II1198::*gat-pheS*	GS_R $cPhe_S$
BP1026B_II1198::*FRT*	GS_S $cPhe_R$
BP1026B_II1198::*FRT pAM3GIQ-3xTY1-BP1026B_II1198*	GS_R $cPhe_R$

$GS_{R/S}$-Resistance/Sensitivity to growth on glyphosate (0.3%)
$cPhe_{R/S}$-Resistance/Sensitivity to growth on chlorinated phenylalanine (0.1%)

b

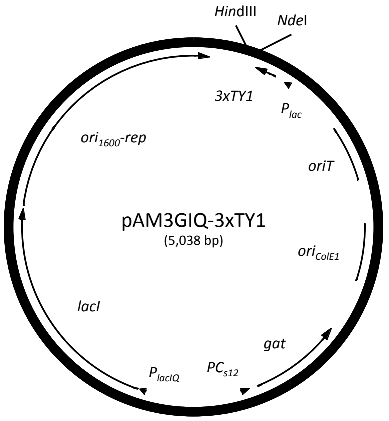

c

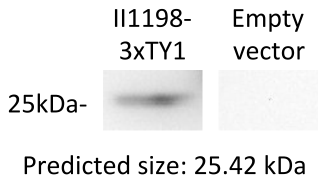

d

Gene	Log_2Fold-Change	
	RNA-seq	RT-PCR
BP1026B_II0683	-1.96	-2.83
BP1026B_I0063	-1.77	-4.35
BP1026B_II0972	-1.40	-2.12

Supplemental Figure 4| BP1026B_II1198 mutant construction, pAM3GIQ-3xTY1 plasmid map, western blot analysis of fusion protein, and validation of RNA-seq. a) Confirmation of phenotypes during the creation of the BP1026B_II1198 mutant and the complemented strain carrying the pAM3GIQ-3xTY1-BP1026B_II1198 plasmid. b) Plasmid map of pAM3GIQ-3xTY1. Abbreviations and features: *Hin*dIII and *Nde*I, restriction enzymes used for cloning of BP1026B_II1198 fusion protein; *3xTY1*, fusion tag; P_{lac}, IPTG inducible promotor; *oriT*, plasmid RP4 origin of transfer for conjugation; ori_{ColE1}, ColE1 origin of replication; *gat*, glyphosate acetyl transferase for glyphosate resistance; PC_{S12}, constitutive promoter for *B. pseudomallei*; P_{lacIQ}, *lacIQ* promotor; *lacI*, *lac* repressor; ori_{1600}*-rep*, *Burkholderia* broad-host-range origin of replication. c) Expression of BP1026B_II1198-3xTY1 protein from pAM3GIQ-3xTY1-BP1026B_II1198 detected by western blot. d) Validation of RNA-seq data through RT-qPCR.
