# Supplementary material for: Identification of a PadR-type regulator essential for intracellular pathogenesis of Burkholderia pseudomallei
Source: Sci Rep. 2021 May 17;11:10405. doi: 10.1038/s41598-021-89852-7 (PMC8128862; doi:10.1038/s41598-021-89852-7)
Supplement: Supplementary file 3 — Supplementary Information 3. [file 41598_2021_89852_MOESM3_ESM.pdf]

Supplemental Table 2: Oligo sequences for RT-qPCR

Gene	Forward	Reverse
BP1026B_I0774	GGG CAT CCG CAA TCT GT	AGC GTG ATT GTG CGT CAT
BP1026B_II1384	ATG ACG ACG CTG AAA CAG A	GAG GCT CTT CAG ATC GTA GTT G
BP1026B_II0521	AAC GTA TCC AAG CTC AAG GG	CGG ATT CCT GCC CAT GTA TT
BP1026B_II0683	CTG GGC GCT CGA CTA TTT	CAT CTG CGC GAA CGA GTA
BP1026B_I0063	GCT CAA ATA CCG CAA ACA GC	CGG AAC TGC TCG TGT TCT T
BP1026B_II0972	GAA TCT TTG CAG TCC GAC AC	GCT CCG AGG ACA GGT AAC
